# Insights into incisional hernia: Factors, symptoms and treatment options

**DOI:** 10.6026/973206300211217

**Published:** 2025-05-31

**Authors:** Patel Visarg Pravinkumar, Swapnil Saraiya, Vivek Amritbhai Patel, Nilam V. Patel, Arifa Bakerywala, Heena Shaikh

**Affiliations:** 1 Department of Surgery, Government Medical College, Baroda, Gujarat, India; 2 Department of General Surgery, University Hospital of North Tees and Hartlepool NHS Foundation Trust, Stockton-on-Tees, England; 3 Department of Orthopedics, Gujarat Adani Institute of Medical Sciences, Bhuj, Kachchh, Gujarat, India; 4 Department of Microbiology, Gujarat Adani Institute of Medical Sciences, Bhuj, Kachchh, Gujarat, India; 5 Department of Pediatric dentistry, Clinical Instructor, Tufts University School of Dental Medicine, Kneeland Street, Boston, Massachusetts; 6 Department of Hospital & Healthcare Management, Masters in Healthcare Leadership, Canada, India

**Keywords:** Incisional hernia, infraumbilical midline incision, prolene mesh, wound seroma

## Abstract

There is a need examine incisional hernia causes; post-operative problems, surgical repair approaches and outcomes. Thirty patients with incisional hernias took part in the study. An additional analysis was conducted on the collected data to identify factors that may contribute, evaluate the effectiveness of different surgical techniques, assess any complications that arose, focus post-operative care and assess surgical outcomes. In six of the cases, there was a wound infection, while in ten of the cases, there was wound dehiscence. Data enlightens us on the problem of incisional hernia, offering how-to guidance in terms of possible conduct in clinical practice and how a patient can be optimally helped.

## Background:

Patients requiring abdominal surgery have a range of surgical options available to them. These include open surgery, laparoscopic surgery, or surgery through a natural orifice. For accessing the abdomen, the midline incision is a commonly chosen option in open surgery. After the surgery is completed, the skilled medical team will carefully sew up the abdominal wall to ensure that the abdominal contents are securely held in place. Once the abdomen is closed, the surgeon may opt to separately close the subcutaneous fat layer before suturing or stapling the skin [1, 2]. When the closure of the abdominal wall is not successful, it can lead to abdominal contents protruding through the defect, which can cause the formation of a bulge and potential symptoms. According to the European Hernia Society, a failure or incisional hernia (IH) is when there is a gap in the abdominal wall, possibly with a bulge near a surgical scar. One way to identify this is by conducting a clinical examination and/or using imaging techniques [3, 4- 5]. Many factors can contribute to the occurrence of IH. Factors that can influence the outcome of a surgery include the specific type of procedure, the underlying health issue being addressed, how long the surgery takes and the specific technique used the patient's age and individual characteristics, any existing medical conditions and whether any complications arise after the surgery. It is important to highlight those individuals who are significantly overweight face a greater risk of developing IH. When evaluating incisional hernias, it is important to confirm the diagnosis, assess the size of the defect, identify the herniated content and evaluate the condition of the abdominal cavity. This thorough assessment aids in determining the most suitable surgical approach for intricate hernias. CT scan imaging is an invaluable tool for obtaining these details [6, 7]. Many studies have identified pre-surgery incisional hernia risk factors. Additionally, researchers have examined the benefits of alternate closure procedures or prophylactic mesh for these patients. Clinicians can assess patient risk with incisional hernia risk-predictive tools like Basta *et al.*'s model. This information can help determine whether prophylactic mesh is necessary for high-risk cases. By implementing these measures, the occurrence of incisional hernia can be decreased, leading to a decreased financial strain on healthcare services [8, 9-10]. Therefore, it is of interest to examine incisional hernia causes, post-operative problems, surgical repair approaches and outcomes. Incisional hernias are complicated, therefore understanding and treating them is essential.

## Materials and Methods:

It was conducted based on the general surgery department of a medical college and hospital. This was an observational study done on 30 patients diagnosed with incisional hernia. At the time of recruitment, all patients were provided with detailed and comprehensive information about the research and informed consent was obtained from them. Inclusion criteria included patients who were older than 14 years, diagnosed with an incisional hernia and had previous abdominal surgery. The excluded criterion was patients under 14 years old and those with non- incisional hernias.

## Sources of data and variables:

The study examined every patient admitted to surgical wards in different units to determine abdominal wall defect causes and variables. The goal of this thorough evaluation was to identify the specific type of hernia and uncover the root cause of the condition. This was achieved by gathering thorough case histories and conducting extensive clinical examinations. All necessary investigations were conducted following a predetermined procedure. After conducting thorough physical examinations on each patient, we were able to make clinical diagnoses, considering any factors that may have contributed to their condition. Individual considerations, including surgical demands, determined the repair approach for each patient. Before undergoing surgical procedures, thorough preparations were made to ensure the patients' optimal health and readiness for the operation. A total of thirty cases were included in the study. The patient's condition was closely monitored for any potential complications, both immediately after the surgery and during the recovery period. To identify contributing factors, evaluate the effectiveness of different surgical techniques, assess complications, emphasise post-operative care and determine surgical procedure results, the data was analysed again.

## Statistical analysis:

Statistical analysis was conducted to evaluate the distribution and outcomes of incisional hernia repairs. Patient distribution and complication rates among the patients were performed with descriptive statistics, means, standard deviations and percentages for categorical variables such as type of incisions, surgical techniques and post-operative complications.

## Results:

The mean age of the patients was 40.5 years, with a standard deviation of 16.33 years, indicating variability in the age of patients. Most of the patients (53.33%) fell within the age group of 31-50 years, while 40% were between 51 and 70 years old. Table 1 illustrates the distribution of the types of incisions used in the surgical procedures. The infraumbilical midline incision was the most common, performed in 53.33% of the cases. The remaining 46.67% of surgeries involved other incisions, indicating a clear preference for the infraumbilical midline incision in this patient group. Wound dehiscence was the most frequent complication, affecting 33.33% of the patients, followed by wound infections in 20% of the cases. These postoperative complications were significant and occurred in a notable portion of the patient population, underscoring the risks associated with these types of surgeries. Table 2 outlines the surgical techniques used for hernia repair in the study. The only mesh repair was used in most cases (80%), while the anatomical repair was performed in 20% of the patients. This shows a clear preference for mesh repair techniques over anatomical repairs in treating incisional hernias in this cohort.

## Discussion:

Over the past decade, there have been notable advancements in the prevention of IH. Although prior suture approaches have reduced frequency, IH remains the most common postoperative complication after abdominal surgery. Obesity and aortic aneurysm increase the incidence of IH, regardless of suture technique. Prophylactic or primary mesh augmentation (PMA) after surgery closure may avoid IH by strengthening the abdominal wall. Several trials have recently been published that examine PMA, but there are still lingering questions about its effectiveness, the optimal technique for performing PMA and the potential postoperative complications [11- 12]. Incisional hernia is a fairly common complication that can occur due to a variety of factors related to the patient and the wound, even when skilled surgeons use proper surgical techniques. It's worth noting the significance of this issue. Research has uncovered different rates of complications, including the formation of seromas and infections at the surgical site, in individuals who undergo hernia repair [13-14]. Clinical practice and patient care are improved by this study on incisional hernias. These findings match hernia repair research. The study conducted by Usher highlights the impressive success rate of polypropylene mesh repair in preventing hernia recurrence. In their research, they found that none of the 48 patients experienced a recurrence, further emphasizing the effectiveness of this approach [15]. Mesh surgery consistently reduces hernia recurrence better than anatomical repair, according to multiple studies. Preventing an incisional hernia is of utmost importance in abdominal surgery. By adhering to specific guidelines and suggestions, you can significantly decrease the likelihood of developing incisional hernias. Here are some suggestions: It is recommended to use slowly absorbing monofilament suture and aim for a suture to wound length ratio of at least 4:1 in order to minimise the risk of incisional hernias, focus on preventing infections, use techniques that are gentle on the tissues and prioritise optimal postoperative patient care. For certain patients and cases with higher risk factors, the rate of incisional hernias can be unusually high even when the closure is done properly. Open abdominal aortic aneurysm (AAA) repair and permanent colostomies increase the likelihood of incisional/parastomal hernias. To prevent this, mesh augmentation is used.

## Limitations:

There were several limitations in this study. Laboratory tests for the diagnosis of complications resulting from incisional hernia were not conducted and the assessment was purely clinical. Also, the report lacked a follow-up assessment to prove the long-term efficacy of the surgical repairs or the hernia recurrence and complications such as seromas or infections. Lastly, the small sample of 30 patients would limit generalizability to a larger population.

## Conclusion:

Data shows valuable information on incisional hernias, gives direction for practice in clinical medicine and improves patient care. In many reports, mesh surgery consistently lowers the recurrence rate of hernias than anatomical repair. Mesh repair recurs less often than anatomical repair; hence, it is the preferred treatment in most scenarios. Laparoscopic correction is recommended for incisional hernias that recur.

## Source of Funding:

There was no financial support concerning this work.

## Figures and Tables

**Table 1:** Type of incision

**Incision Type**	**Number of Patients**	**Percentage (%)**
Infraumbilical Midline	16	53.33%
Other Incision Types	14	46.67%
Total	30	100%

**Table 2:** Methods of surgery employed

**Repair Type**	**Number of Patients**	**Percentage (%)**
Anatomical Repair	6	20.00%
Onlay Mesh	24	80.00%
Total	30	100%
